# Novel SNP markers for flowering and seed quality traits in faba bean (*Vicia faba* L.): characterization and GWAS of a diversity panel

**DOI:** 10.3389/fpls.2024.1348014

**Published:** 2024-03-06

**Authors:** Hannah Ohm, Johanna Åstrand, Alf Ceplitis, Diana Bengtsson, Cecilia Hammenhag, Aakash Chawade, Åsa Grimberg

**Affiliations:** ^1^ Department of Plant Breeding, Swedish University of Agricultural Sciences (SLU), Lomma, Sweden; ^2^ Lantmännen Agriculture, Plant Breeding, Svalöv, Sweden

**Keywords:** *Vicia faba* (faba bean), diversity panel, GWAS (genome wide association study), DArT-seq, field trial, SNPs (single nucleotide polymorphism), flowering

## Abstract

Faba bean (*Vicia faba* L.) is a legume crop grown in diverse climates worldwide. It has a high potential for increased cultivation to meet the need for more plant-based proteins in human diets, a prerequisite for a more sustainable food production system. Characterization of diversity panels of crops can identify variation in and genetic markers for target traits of interest for plant breeding. In this work, we collected a diversity panel of 220 accessions of faba bean from around the world consisting of gene bank material and commercially available cultivars. The aims of this study were to quantify the phenotypic diversity in target traits to analyze the impact of breeding on these traits, and to identify genetic markers associated with traits through a genome-wide association study (GWAS). Characterization under field conditions at Nordic latitude across two years revealed a large genotypic variation and high broad-sense heritability for eleven agronomic and seed quality traits. Pairwise correlations showed that seed yield was positively correlated to plant height, number of seeds per plant, and days to maturity. Further, susceptibility to bean weevil damage was significantly higher for early flowering accessions and accessions with larger seeds. In this study, no yield penalty was found for higher seed protein content, but protein content was negatively correlated to starch content. Our results showed that while breeding advances in faba bean germplasm have resulted in increased yields and number of seeds per plant, they have also led to a selection pressure towards delayed onset of flowering and maturity. DArTseq genotyping identified 6,606 single nucleotide polymorphisms (SNPs) by alignment to the faba bean reference genome. These SNPs were used in a GWAS, revealing 51 novel SNP markers significantly associated with ten of the assessed traits. Three markers for days to flowering were found in predicted genes encoding proteins for which homologs in other plant species regulate flowering. Altogether, this work enriches the growing pool of phenotypic and genotypic data on faba bean as a valuable resource for developing efficient breeding strategies to expand crop cultivation.

## 1 Introduction

Given the mounting environmental concerns regarding climate change and planetary boundaries, developing a more sustainable agriculture and food production system is an urgent global goal (Steffen et al., 2015). Legumes play an important role in promoting sustainable agriculture due to their agronomic, environmental, and nutritional benefits (Voisin et al., 2014; Rubiales et al., 2021). Cultivation of legumes is beneficial in crop rotations due to their capacity to fix atmospheric nitrogen through symbiosis with soil bacteria, replacing the need for synthetic nitrogen fertilizer. In addition, legumes break the disease cycles in cereal-dense agricultures (Rubiales et al., 2021), promoting reduced pesticide use. Due to their nutritional benefits and high protein content, legumes could replace animal-based protein in human diets and are therefore considered a prerequisite for transitioning to a more sustainable food system (Willett et al., 2019). However, only a small number of pulse crops are currently contributing to the world’s food production among which soybean (*Glycine max*) accounts for 80% of the total legume production (*FAOSTAT, 2021*). To promote the cultivation and consumption of a diverse array of legumes, it is essential to provide growers with cultivars that exhibit high and stable yields and seed qualities valued by food producers. Therefore, there is an urgent demand for increased plant breeding efforts on pulse crops.

Faba bean [*Vicia faba* L. (Fabaceae)] is a leguminous crop characterized by seeds with a relatively high protein content (30%), and holds a high potential for increased cultivation and use in food. However, current challenges to its cultivation and use are unstable yields, disease susceptibility, sensitivity to drought, antinutritional compounds such as tannins, as well as convicin and vicin that cause favism in individuals with a specific hereditary disease (Maalouf et al., 2018; Björnsdotter et al., 2021). Further, it is a partially allogamous species with outcrossing rates between 4% and 84% (Ellwood et al., 2008). While no wild ancestors have yet been found, over 38,000 accessions of *V. faba* are available in genebanks worldwide, represented in collections by cultivated forms only (Duc et al., 2010). Even though a wide diversity of plant and seed phenotypes has been reported (Maalouf et al., 2018), the scarce phenotypic and genotypic characterization of germplasm collections limits the exploitation of available diversity as a resource for breeding.

Faba bean has one of the largest genomes of the diploid crops, with approximately 13 Gb distributed across six pairs of chromosomes, and possibly around 85% composed of repetitive DNA (Khazaei et al., 2021). Detailed genetic and physical maps of markers and quantitative trait loci (QTL) have previously not been available for faba bean. However, several genetic consensus maps based on biparental populations and single nucleotide polymorphism (SNP) markers identified from transcriptome data, together with genotyping-by-sequencing (GBS) markers mapped to publicly available faba bean genomic and transcriptomic sequences, have been developed lately (Webb et al., 2016; Carrillo-Perdomo et al., 2020; Wang et al., 2021; Abou-Khater et al., 2022; Maalouf et al., 2022; Li et al., 2023; Zhao et al., 2023). Recently, a significant milestone was achieved with the publication of the first reference genome of faba bean, providing a valuable resource for further genetic analysis (Jayakodi et al., 2023).

Genome-wide association study (GWAS) has become an important tool for geneticists in identifying genomic loci governing target traits in plants and is based on the association of genotypic and phenotypic data (Torkamaneh and Belzile, 2022). Through GWAS on faba bean, genetic markers have been associated with agronomic traits such as heat stress and herbicide tolerance, plant architecture (height, branching, and flower/pod placement), time to flowering, and seed yield (Abou-Khater et al., 2022; Maalouf et al., 2022; Karaköy et al., 2023; Li et al., 2023). Several GWAS on faba bean with a focus on seed quality aspects were also recently published, making use of the available reference genome to localize markers. Jayakodi et al. (2023) identified genetic markers for seed size based on a 90K SPET genotyping assay on a diversity collection of 197 accessions, and Skovbjerg et al. (2023) genotyped a 7-parent MAGIC population with a 21,345 SNP array which allowed for the identification of 238 markers for agronomic and seed quality traits. Further, Zhao et al. (2023) genotyped 121 individuals from an F2 population using a 130K SNP chip which was developed from RNA-seq data on flowering and leaf tissues (Wang et al., 2021), which allowed for the identification of markers associated with 65 seed traits, including seed quality.

To date, only a limited number of GWAS on faba bean have utilized diversity panels, and none of them have considered both agronomic and seed quality traits. In this study, we assembled a diversity panel of 220 accessions of faba bean originating from diverse geographical regions, sourced from gene bank material and commercially available varieties. The aims of this study were to i) characterize this diversity panel for agronomic- and seed quality traits in field trials at Northern latitudes, ii) analyze the effects of selection pressure through breeding on faba bean to date, and iii) to identify genetic markers (SNPs) associated with target traits through GWAS.

## 2 Materials and methods

### 2.1 Plant material

The diversity panel in this study comprised 220 faba bean accessions selected to represent a wide variation of geographical origin, flower and seed color, seed size, plant height, tannin and convicine/vicine content, and breeding advancement status. The sources of seeds were genebanks (Nordic Genetic Resource Centre (SWE054, https://www.nordgen.org/en/), Genebank of Leibniz Institute of Plant Genetics and Crop Plant Research (DEU146, https://www.ipk-gatersleben.de/), and National Plant Germplasm System of the United States Department of Agriculture (USA022, https://npgsweb.ars-grin.gov/gringlobal/search) and commercially available varieties from breeders in Europe. The country of origin for each accession and link to the source of seeds are found in **Supplementary Datasheet 1**. A heat map of the distribution of the accessions based on origin was generated using the ggplot2 package in R (Wickham, 2016).

Based on passport data, the 220 accessions from the diversity panel were classified into four categories with increasing level of advancement. The categories were *cultivated* (plant material with scarce information annotated by gene banks as cultivated, unknown, or ‘wild’), *landraces* (plant material annotated either as landrace by gene banks or known heirloom cultivars, including accessions from a seed preservation and collection program in Sweden), *advanced* (plant material that has undergone some level of advancement through either research or breeding but not registered as a variety in the European Community Plant Variety Office database (CPVO) as per 2022-10-14) and *varieties* (varieties registered in the CPVO database as per 2022-10-14, including both agricultural and vegetable varieties). The term *cultivated* is considered as the least developed plant material.

Seeds from the 220 accessions were amplified in the field or greenhouse during 2020. The field site for seed amplification was situated at 55.90_N, 13.09_E, where the accessions with small- to medium-sized seeds were sown by machine, and accessions with larger seeds were sown manually. Sowing was conducted in mid-April and manual harvesting occurred at maturity from late July to August. Seed amplification in the greenhouse was conducted between November 2020 to February 2021 by growing single plants in 7.5 L pots in soil fertilized with 3M Plus Basacote (Compo Expert, Muenster, Germany). The temperatures were 21°C (day) and 18°C (night), at a relative humidity of 70%, with a 16 h photoperiod, supplemented with Son-T PIA 400 W sodium lamps (Philips, Amsterdam, Netherlands) when the natural light fell below 200 µmol · m^-2^ s^−1^ photosynthetically active radiation. Plants were isolated during flowering either by spatial separation (in the greenhouse) or with perforated plastic isolation bags (in the field) to prevent cross-pollination between accessions. Pods were manually harvested, threshed and stored at 4°C.

### 2.2 Field trial design

The field experiments were carried out at the SITES Research Station Lönnstorp, SLU, in Alnarp (55.65_ N, 13.06_ E) spanning two consecutive growing seasons in 2021 and 2022, in an alpha lattice design with 11 blocks and two replicates. The field was fertilized prior to sowing (300 kg/ha Pk11-21 in 2021, 350 kg/ha Pk11-21 in 2022). Fifty seeds were hand-sown in each plot à 1 m x 0.75 m, resulting in a density of 66 seeds/m^2^ and an average seed distance of approximately 15 cm. Seeds used in the 2021 field trial derived from seed amplification conducted during 2020, whereas seeds for the 2022 field trial derived from the harvest of the 2021 field trial. During both trials, the field was covered with a fiber cloth until plant establishment, to protect the germinated seedlings from birds. The field was watered as needed (four times early in the growth season of 2021 to secure seedling establishment, none in 2022), manually weeded between plots, and sprayed against weeds (Corum^®^ once in 2021, Corum^®^ and Dash^®^ once in 2022) and aphids (MAVRIK^®^ AQUAFLO once in 2021, Teppeki^®^ once in 2022). For detailed weather data, soil conditions, and further information on field management, see **Supplementary Datasheet 1**.

### 2.3 Phenotyping of agronomic and seed quality traits

Field phenotypic data were collected for the following parameters: establishment (number of established plants per plot ~35 days after sowing), plant height (average height of five randomly selected plants/plot measured at ~75 days after sowing), days from sowing to flowering (when 50% or more of the established plants in a plot had at least one open flower), days from sowing to maturity (when 50% or more of the established plants in a plot had filled, dry and brown/black pods). The damage caused by the broad bean weevil (*Bruchus rufimanus* Boh.) and its parasitoids was assessed after harvest by classifying 100 randomly selected seeds as healthy or infested seeds as described by Carrillo-Perdomo et al. (2019). The testa color of the seeds was determined by classifying them into four color categories (dark, red, green, and light). The gradients of reference colors for each category were defined by digitally sampling photos of seeds with a color picker tool using Affinity Photo software (**Supplementary Figure 1**). Seed dimensions (length, width, and area) and thousand-grain weight (TGW) were determined using 100-200 seeds with the seed analyzer MARViN ProLine I (Marvitech, Germany). The yield parameter expressed as gram seeds per plant (YIELD) was determined by dividing the total weight of harvested seeds from a plot (g) by the number of established plants in that plot. The total number of seeds per plant (SEEDS) was determined by dividing the total weight of harvested seeds from a plot by its TGW, then multiplying by 1000, and finally dividing by the number of established plants in that plot.

### 2.4 Seed protein and starch analysis

For protein and starch analysis, approximately 5 g seeds (at least 10 seeds) from each of the 220 faba bean accessions from the 2021 field trial were ground into flour using a centrifugal mill at 10,000 rpm and passed through a 0.5 mm sieve (ZM 200, Retsch GmbH Haan, Germany) and subsequently freeze-dried for 48 h at -60°C. Raw protein content was analyzed using the Dumas method (N×6.25) on 0.5 g flour (Eurofins Food & Feed Testing Sweden AB, Lidköping, Sweden).

Total starch content was determined using the Megazyme Total Starch Assay Kit K-TSTA (Megazyme, Bray, Ireland) on 80 mg flour. The assay is based on starch degradation with α-amylase and amyloglucosidase, followed by colorimetric determination of the released glucose. To ensure accurate starch determination and minimize interference, any potential presence of free sugars in the flour was extracted with ethanol washes prior to the starch analysis. Thereafter, the recommended procedure was followed, with the exception of a downscaled enzymatic colorimetric conversion that facilitated more convenient handling of the samples. The absorbance was measured at 510 nm on a microplate spectrophotometer (Multiskan GO, ThermoFisher Scientific, MA, USA).

### 2.5 Statistical analysis of the phenotypic data

The best linear unbiased estimates (BLUE) values, broad-sense heritability, and variance components of each trait were obtained using the META-R software with adjustments following the alpha lattice design (Alvarado et al., 2020). The model used for the calculation of the BLUE values was:


$$Y_{ijkl}=\mu +Loc_{i}+Rep_{j}(Loc_{i})+Block_{k}(Loc_{i}Rep_{j})+Gen_{l}+Loc_{i}\times Gen_{l}+\varepsilon_{ijkl}$$

where Y*_ijkl_* is the trait of interest, *μ* is the overall mean effect, Loc*_i_* is the effect of the *i*th location, Rep*_j_* (Loc*_i_*) is the effect of the *j*th replicate in the *i*th location, Block*_k_* (Loc*_i_* Rep*_j_*) is the effect of the *k*th block within the *i*th location and the *j*th replicate, Gen*_l_* is the effect of the *l*th genotype, the Loc*_i_* ×Gen*_l_* is the effect of the environment × genotype interaction, and ε*_ijkl_* is the effect of the error associated with the *i*th location, the *j*th replication, the *k*th block, and the *l*th genotype, as specified in Alvarado et al. (2020). The BLUE values for each trait of all accessions were obtained from two replicates from each year (with the exception of protein content, starch content, and bean weevil damage, for which only one year of data was available) by assuming fixed genotypic and random environmental effects. Broad-sense heritability was calculated using:


$$H^{2}=\frac{\sigma_{g}^{2}}{\sigma_{g}^{2}+\frac{\sigma_{ge}^{2}}{nLoc}+\frac{\sigma_{e}^{2}}{nLoc\times nRep}}$$

where $\sigma_{g}^{2}$ , $\sigma_{e}^{2}$ , and $\sigma_{ge}^{2}$ are the genotype, error, and genotype by environment interaction variance components, respectively, nLoc is the number of environments, and nRep is the number of replicates, as in Alvarado et al. (2020). The BLUE value of each accession was used for linear pairwise correlations for each trait and were assessed using Pearson’s correlation coefficient, with an ANOVA t-test where p< 0.001: ***, p< 0.01: **, p< 0.05: *. Differences between the groups, defined by the different levels of advancement, were estimated using a pairwise t-test with Bonferroni adjustment.

### 2.6 DNA extraction and DArTseq genotyping

Leaf tissue for DNA extraction was sampled from greenhouse-grown plants in March 2022. Seeds from each accession in the diversity panel (from isolated plants 2020, see above) were sown in 2 L pots filled with soil (50% peat, pH 5.5-6.5, added per m^3^ soil: 5.5 kg lime, NPK 11-5-18 kg, 200 g micronutrients and 100 g iron). Greenhouse parameters were as described above. Two weeks after germination, a single true leaf from five plants per accession was sampled and put in a petri dish on ice. Two 3 mm diameter discs were punched from each of the five leaves, resulting in a total of ten discs per accession. The discs were pooled and placed in a single well of a 96-well plate on ice. For eight of the accessions less than five plants were available, and the sampling was therefore evenly distributed among the available plants to obtain ten discs. The puncher and punching mat were carefully cleaned between sampling of each accession, to prevent cross-contamination. The leaf tissues were freeze-dried for 40 h at -60°C and then kept at room temperature until DNA extraction. In total, 187 of the 220 accessions from the diversity panel were sampled. DNA extraction was performed by Intertek ScanBi Diagnostics (Alnarp, Sweden) using sbeadex™ plant DNA extraction kit (Biosearch Technologies, Hoddesdon, United Kingdom). A high DNA quality was confirmed by checking DNA integrity using agarose (1%) gel electrophoresis. The samples were genotyped by DArTseq (Diversity Array Technologies, Canberra, Australia), which is a genome complexity reduction-based sequencing using restriction enzymes. The sequencing depth used was 800 000 counts/sample.

### 2.7 Marker filtering and sequence mapping to the reference genome

Through DArTseq 19,770 SNP markers were identified, and their flanking sequences were aligned using BLAST+ to the reference genome of *Vicia faba* ‘Hedin/2’ (Jayakodi et al., 2023) to determine their respective positions within the genome. The aligned markers were filtered for >95% replication average and a call rate >50%. Imputation of the missing data in the remaining markers was conducted in TASSEL 5.0 (Bradbury et al., 2007) using the five closest neighboring accessions and Eucledian distance. A subsequent filtering step was applied to select markers with only one aligned position in the *V. faba* (‘Hedin/2’) reference genome and with an aligned sequence having an E-value >10^-6^, resulting in the identification of 6,606 markers in the *V. faba* genome. Phylogenetic distance between the accessions was estimated using neighboring clustering in TASSEL 5.0 (Bradbury et al., 2007) and visualized with Interactive tree of life (iTOL) (Letunic and Bork, 2021). SNP density was visualized using the rMVP R package (Yin et al., 2021). Linkage disequilibrium was calculated with the imputed genotype data in TASSEL 5.0 (Bradbury et al., 2007) and visualized in R 4.2.2 using script written by Mohsin (2021) based on Remington et al. (2001).

### 2.8 Genome-wide association study

A GWAS was conducted for each trait using the Genome Association and Prediction Integrated Tool (GAPIT) 3.0 function (Wang and Zhang, 2021) implemented in R 4.2. Principal component analysis (PCA) and the VanRaden kinship matrix were conducted using the GAPIT 3.0 function to determine any underlying population structure. The GWAS was conducted for each trait separately using the Bayesian-information and Linkage-disequilibrium Iteratively Nested Keyway (BLINK) model implemented in the GAPIT 3.0 function, with zero principal components and a minor allele frequency (MAF) threshold set to 0.01. Predicted genes in which the associated markers were localized were annotated based on the faba bean reference genome, as well as on the highest sequence similarity of legume species using blastX at NCBI using default settings (BLAST).

## 3 Results

### 3.1 The diversity panel

**Figure 1:**
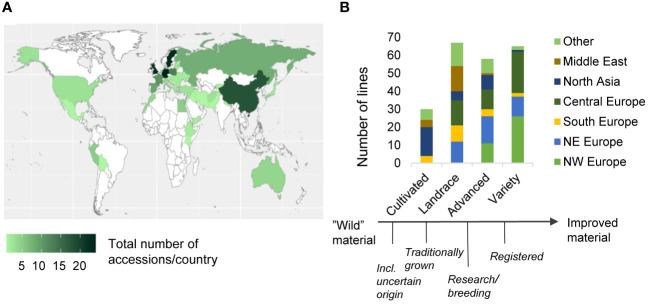
Overview of the diversity panels' geographical origin and breeding advancement status, and genetic relatedness. Distribution of the accessions’ geographical origin per country **(A)**. Geographical distribution of the accessions per country, sorted on their breeding advancement **(B)**. For the definitions of the categories, see Materials & Methods.

The diversity panel had a broad geographical distribution with representatives from all continents, with a majority of the accessions originating from northern and central Europe (34% and 23%, respectively) and North Asia (14%) (**Figure 1A**). Based on their origin and passport data (**Supplementary Datasheet 1**), the 220 accessions from the diversity panel were classified into four categories with increasing degree of advancement (**Figure 1B**). All four categories were represented by approximately 60 accessions, except for the least advanced category, which comprised only 30 accessions. The two most advanced categories were primarily composed of accessions of European origin, while the category “landrace” included accessions from all geographical regions. The category “cultivated” on the other hand, were predominantly composed of non-European material.

### 3.2 Phenotypic diversity and correlations

**Figure 2:**
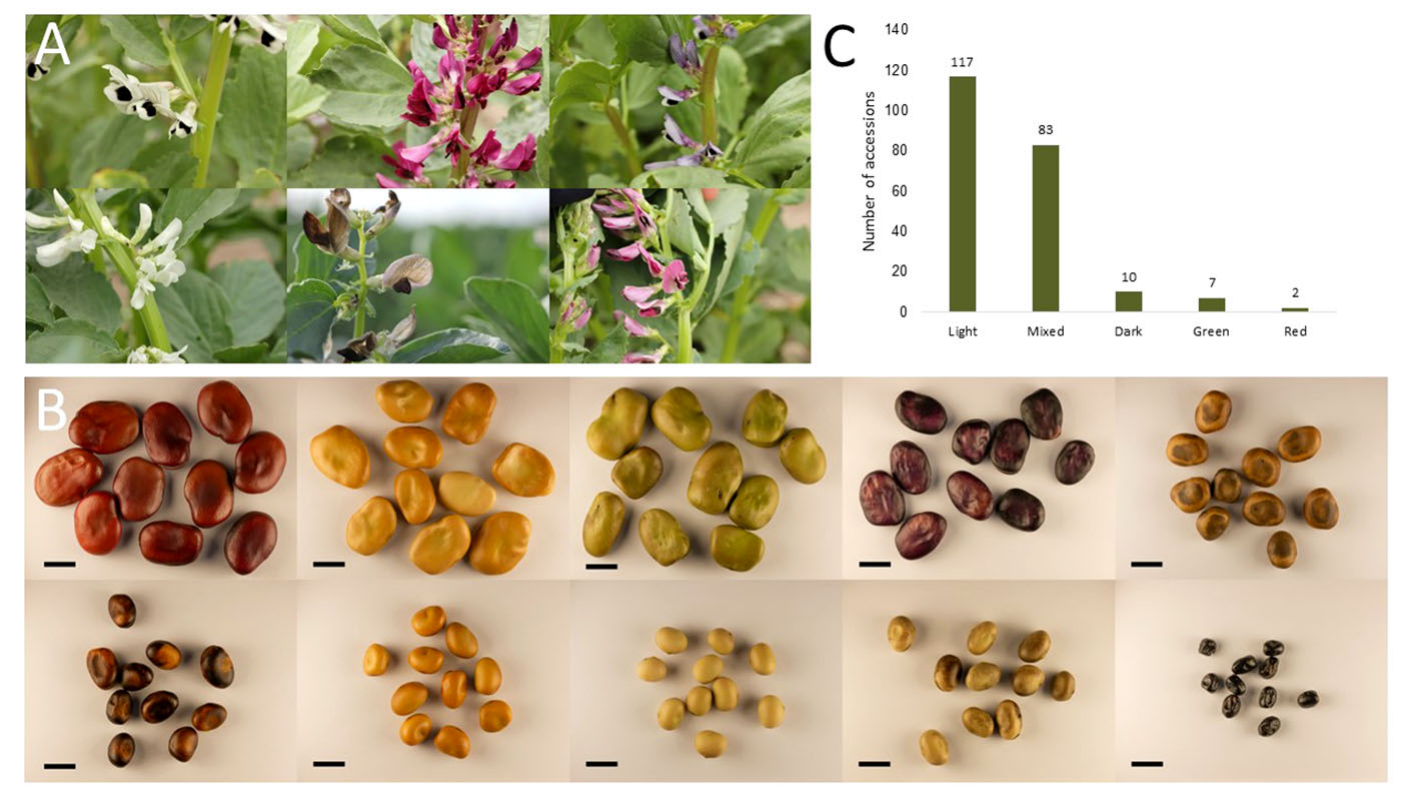
Examples of variation in flower color **(A)** and seed color, shape, and size **(B)** present in the diversity panel used in this study. Graph **(C)** shows the grouping of the whole diversity panel into distinct seed color categories shown in **Supplementary Figure 1**, with the panels below showing the color scales included in every category. Scale bar in B is 1 cm.

**Figure 3:**
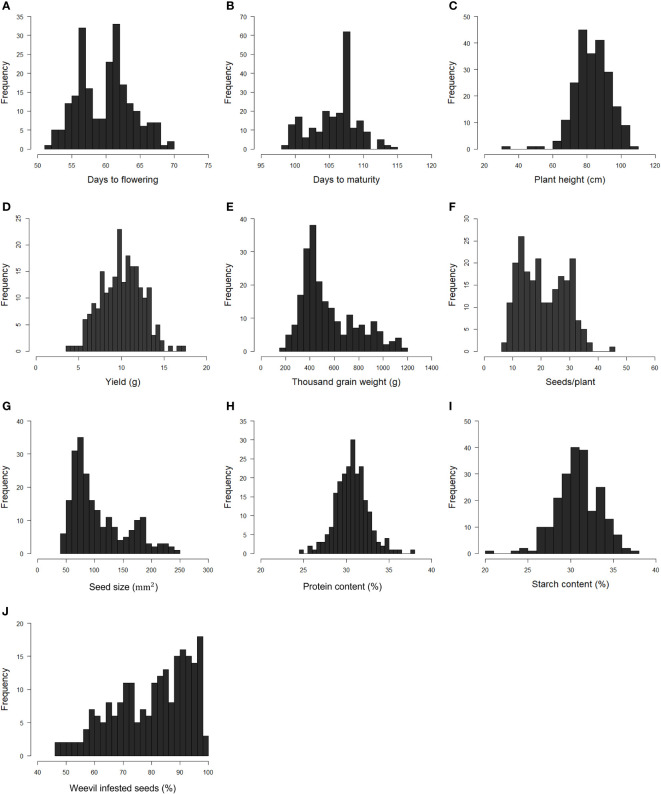
Histograms showing the frequency distribution of Best Linear Unbiased Estimates (BLUE) values of each trait evaluated in the faba bean diversity panel in field trials across two years 2021 and 2022*. Days to flowering **(A)**. Days to maturity **(B)**. Plant height **(C)**. Seed yield per plant **(D)**. Thousand-grain weight **(E)**. Number of seeds per plant **(F)**. Seed size **(G)**. Seed protein content by dry weight **(H)**. Seed starch content by dry weight **(I)**. Proportion of seeds infested by bean weevil **(J)**. *Data for bean weevil, protein content, and starch content are only for the 2021-year field trial.

The phenotypic raw data of the 220 accessions in the diversity panel displayed a broad range of values for several key agronomic traits (**Supplementary Datasheet 1**). Flower colors of brown, white, purple, red, and variegated (i.e., white petals with a black wing melanin spot), as well as various seed shapes and testa colors, were represented (**Figures 2A–C**). In the field trials during 2021 and 2022, the average establishment of the 50 sown seeds was 82-91% approximately 30 days after sowing. The phenotypic raw data was used to estimate the BLUE values for each year (**Supplementary Figure 2**) and for both years (**Figure 3**). Phenotype metrics of BLUE values based on both years’ phenotype data are shown in **Table 1**. All measured traits showed broad-sense heritability values above 0.6 and with the majority substantially exceeding 0.8 (**Table 1**). Time from sowing to flowering in the diversity panel varied between 52-70 days (**Figure 3A**). While most of the accessions flowered between 55-62 days after sowing, six accessions consistently exhibited very early flowering (<55 days) and 17 accessions consistently displayed very late flowering (>66 days) across both years. Time from sowing to maturity varied between 98-114 days, with most accessions maturing at day 108 (**Figure 3B**). At the time point of growth stabilization (~77 days after sowing), the plant height showed a distribution range between 60-106 cm, but one dwarf accession only attained 33 cm (**Figure 3C**). Seed yield (g per plant) showed a substantial variation between accessions (4-17 g/plant), with normal distribution (**Figure 3D**). Twenty-one of the highest-yielding accessions (>12 g/plant) performed consistently across both years. Thousand-grain weight (TGW) varied between small, pea-sized accessions with values below 200 g up to large-seeded accessions at 1200 g (**Figures 2B** and **3E**), which was also reflected in the variation in seed size (i.e., seed area, **Figure 3G**). The number of seeds per plant varied from 7 to 37, with a few outliers reaching as high as 56 (**Figure 3F**). Seed protein and starch content in the diversity panel exhibited substantial variation, with values ranging from approximately 25 to 37% for both traits (**Figures 3H, I**). The level of bean weevil damage was generally high and showed a variation between 45 to 100% infested seeds in the accessions (**Figure 3J**).

**Table 1:** Phenotype metrics, broad-sense heritability (H²), and coefficient of variation (CV) for each trait characterized in the diversity panel consisting of 220 accessions.

	Min	Max	Mean	H²	CV	Accession with min value	Accession with max value
**HEIGHT (cm)**	33.60	106.00	83.16	0.89	6.56	Dwarf Ö53	Ashleigh
**DTF (days)**	52.0	69.5	59.8	0.94	2.28	Felix	CHA CHA
**DTM (days)**	98.25	114.00	105.85	0.78	2.00	ATC 63752	Banquise
**TGW (g)**	155.20	1161.96	549.87	0.97	10.10	Mikko	FAB 590
**YIELD (g/plant)**	3.91	17.08	10.05	0.65	22.38	Mikko	Karmazyn
**SIZE (mm²)**	41.60	248.60	107.10	0.98	7.59	Mikko	Super Aquadulce
**SEED WIDTH (mm)**	6.43	15.50	10.03	0.98	3.60	Mikko	Super Aquadulce
**SEED LENGTH (mm)**	8.35	20.93	13.13	0.98	3.62	Mikko	Grebo
**WEEVIL (% infested seeds)**	47.4	99.3	80.06	0.90	29.54	COLUMBA	Syria local small
**SEEDS (no/plant)**	6.69	44.13	20.72	0.88	23.59	Super Aquadulce	Nanaux
**PROTEIN (%)**	25.05	38.05	30.64	0.71	4.73	ATC 63759	Dwarf Ö53
**STARCH (%)**	20.93	37.84	30.86	0.66	6.63	Dwarf Ö53	Ticol HÖG

Min, max, and mean for each trait are given as the Best Linear Unbiased Estimates (BLUE) values across years. The accessions with the min and max values of each trait are given to the right, for phenotypic data of each accession in the panel see **Supplementary Datasheet 1**. HEIGHT, plant height (cm); DTF, days to flowering; DTM, days to maturity; TGW, thousand grain weight (g); YIELD, seed weight per plant (g); SIZE, seed size as area (mm2); WEEV, proportion of seeds infested by bean weevil (%); SEEDS, number of seeds per plant; PROTEIN, seed protein content by dry weight (%); STARCH, seed starch content by dry weight (%). STARCH, seed starch content by dry weight (%). Data for WEEVIL, PROTEIN, and STARCH is from 2021 only.

### 3.3 Pairwise correlation coefficient analysis of agronomic traits

**Figure 4:**
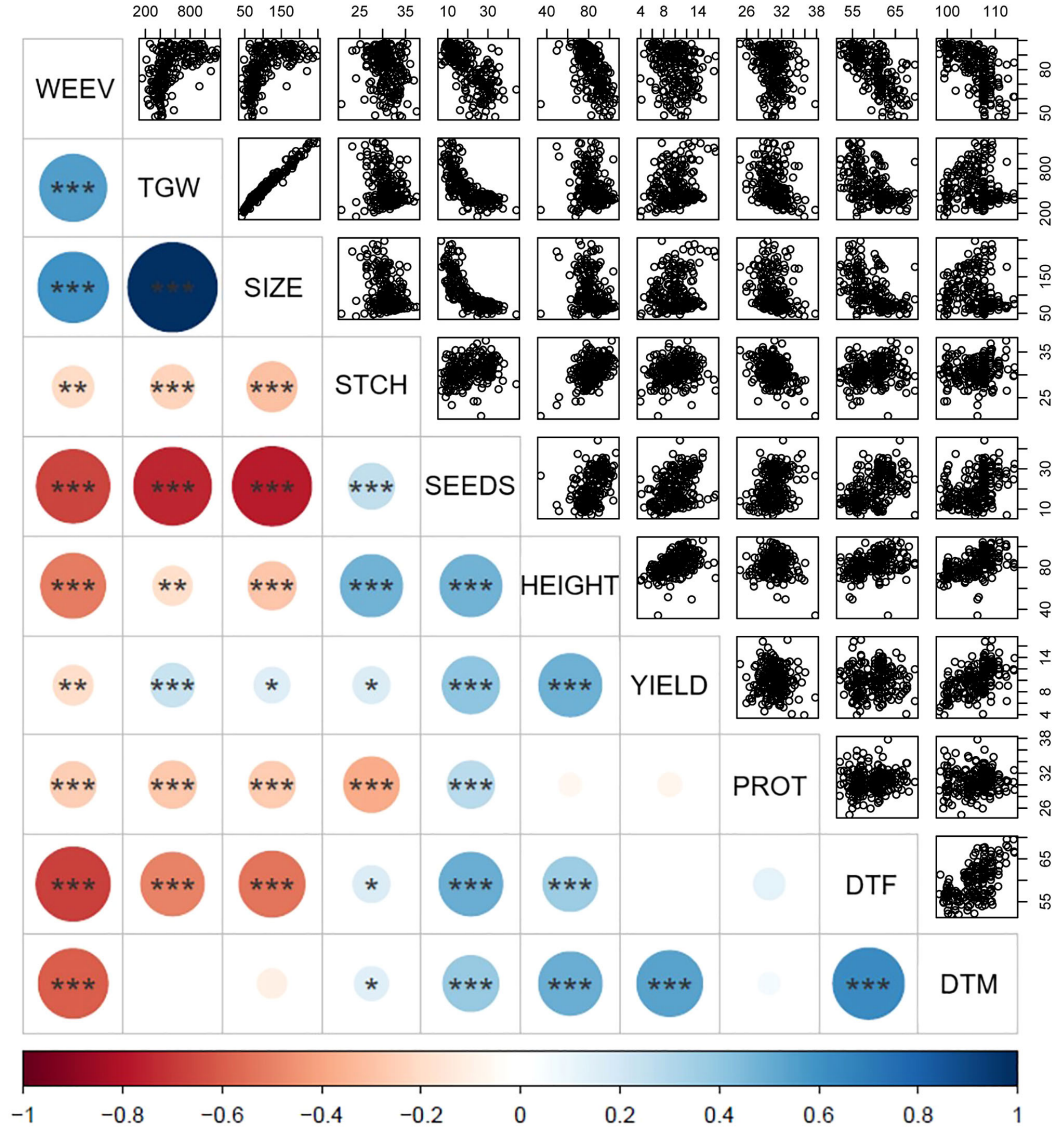
Pairwise correlations between traits characterized in the diversity panel based on phenotypic values. The color intensity and circle size represent the strength of Pearson’s correlation coefficient with larger circle sizes indicating stronger correlation and the color scale indicating negative correlations in red and positive correlations in blue according to the scale at the bottom. Number of stars indicates the significance level based on ANOVA t-test with p < 0.001: ***, p < 0.01: **, p < 0.05: *, and p > 0.05: no star, and correlation values for all traits are found in **Supplementary Table 1**. The scatter plots in the upper panel show all correlated data points. WEEV, proportion of seeds infested by bean weevil (%); TGW, thousand grain weight (g); SIZE, seed size as area (mm²); DTF, days to flowering; DTM, days to maturity; HEIGHT, plant height (cm); YIELD, seed weight per plant (g); SEEDS, number of seeds per plant, PROT, seed protein content by dry weight (%), STCH, seed starch content by dry weight (%).

To examine phenotypic pairwise correlations among the different traits characterized in the diversity panel, a Pearson’s correlation coefficient matrix was computed [**Figure 4** and **Supplementary Table 1** for correlation coefficient values (r)]. Interestingly, several traits showed strong correlations, with all of the mentioned correlations being highly significant at p < 0.01 or lower. The positive correlation found between TGW and seed size confirms the intuitive relationship of larger seeds having a higher weight (r = 0.99). An inverse correlation was observed for seed size and number of seeds per plant (r = -0.77), indicating a common biological trade-off where larger seeds are associated with fewer seeds per plant. Yield was positively correlated with days to maturity (r = 0.53), plant height (r = 0.49), number of seeds per plant (r = 0.40), and moderately with TGW (r = 0.23).

Days to flowering was positively correlated with days to maturity (r = 0.63), indicating a clear relationship where plants that flowered later also matured later. Later flowering plant material was associated with a higher number of seeds per plant (r = 0.50) and taller plants (r = 0.37). Interestingly, the data indicated that yield was independent of days to flowering but positively associated with days to maturation. This indicates that a later maturation is beneficial for a higher yield, whereas later time to flowering is not. Furthermore, days to flowering was negatively correlated with TGW (r = -0.50) and seed size (r = -0.53), indicating that accessions with later flowering tend to have smaller seeds.

Regarding nutritional seed quality traits, a negative correlation was observed between protein content and starch content (r = -0.38). Furthermore, both starch content and protein content were negatively correlated with TGW (r = -0.24 and r = -0.27, respectively) and seed size (r = -0.30 and r = -0.27, respectively). These observations imply that smaller seeds tend to have a higher content of both protein and starch, as compared to larger seeds. Consequently, it can be inferred that the content of other seed constituents is higher in larger seeds. Interestingly, protein content did not show any correlations with yield but a slight positive correlation with number of seeds (r = 0.27). For starch content, there was only a weak positive correlation indicated with yield (r = 0.17, at p< 0.05) but a clear correlation with plant height (r = 0.48).

Bean weevil damage of seeds exhibited a strong negative correlation with days to flowering (r = -0.68) and maturity (r = -0.60), indicating that plants that flowered and matured earlier were more prone to have a higher degree of infested seeds. Correlations were observed for weevil damage with both seed size (r = 0.61) and TGW (r = 0.55), which implied that accessions with larger seeds were more infested. Correlations also showed that increased bean weevil damage was significantly associated with fewer seeds per plant (r = -0.66) and shorter plant height (r = -0.52). Interestingly, the extent of bean weevil damage showed only a slight correlation with lower yields (r = -0.19). A slight negative correlation was found between infested seeds and starch (r = -0.21) and protein levels (r = -0.26).

### 3.4 Distribution of traits depending on advancement status

**Figure 5:**
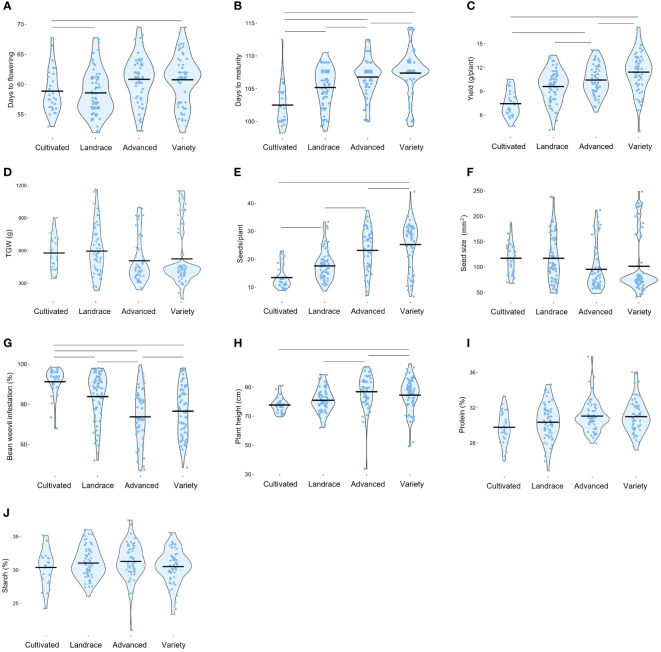
Trait distributions are based on the advancement status of the accessions. Data shown are the Best linear unbiased estimates (BLUE) values of each trait evaluated in the faba bean diversity panel in field trials across two years 2021 and 2022*. Each dot represents one accession. The bold lines in each violin plot are the mean values. Days to flowering **(A)**. Days to maturity **(B)**. Seed yield per plant **(C)**. Thousand-grain weight **(D)**. Number of seeds per plant **(E)**. Seed size as seed area **(F)**. Proportion of seeds infested by bean weevil **(G)**. Plant height **(H)**. Seed protein content by dry weight **(I)**. Seed starch content by dry weight **(J)**. *Data for bean weevil, protein content, and starch content is only for 2021 years' field trial. Lines between groups indicate a significance level of at least p > 0.05. Note that sample sizes differ between groups and that non-significant pairs may be due to differences in statistical power.

The distribution of the agronomic traits, grouped based on their breeding status, is visualized in the violin plots of **Figure 5**. Significant differences were observed between more improved and less improved material, with the former showing an increase in days to flowering and days to maturity. In addition, compared to its less improved counterpart, the more improved material exhibited higher yield, more seeds per plant, and taller plants. Although there was a trend towards decreasing TGW and seed size for more advanced material, these differences did not reach statistical significance. Notably, the level of bean weevil damage was lower on the improved plant material compared to the less improved counterpart. Higher bean weevil damage on the less improved material could potentially be linked to the earlier flowering observed in this category, which is negatively correlated to bean weevil damage (**Figure 4**). However, even though the level of damage on the improved material was lower, more variation was observed within this group as compared to the least improved material. No significant patterns of change for seed protein and starch content through breeding could be observed.

### 3.5 Marker filtration and mapping to reference genome

**Figure 6:**
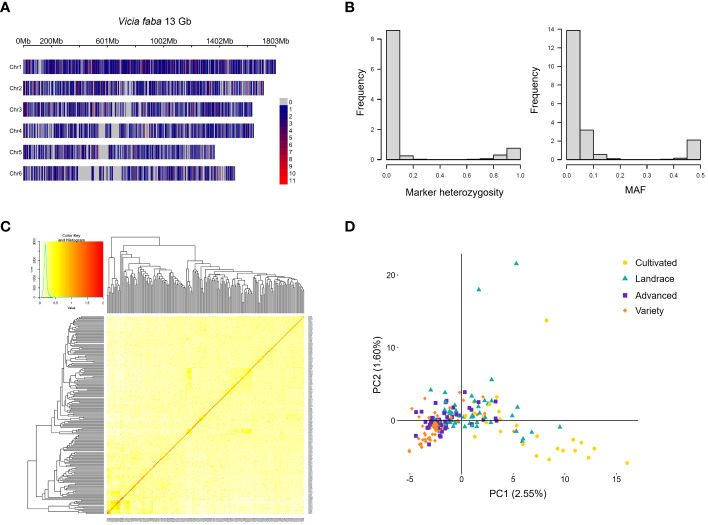
Genetics of the faba bean diversity panel. The markers obtained from DArTseq mapped to the ‘Hedin/2’ reference genome of *V. faba* with the color scale of the gradient indicating the marker density as number of SNPs in a 1Mbp window size **(A)**. Marker heterozygosity and minor allele frequency (MAF) **(B)**. Kinship matrix **(C)**. Principle component (PC) analysis plot based on genotype data and color-coded according to level of advancement **(D)**.

To identify markers in the faba bean genome for genetic analysis, 187 of the accessions in the diversity panel were genotyped using the genotyping by sequencing method DArTSeq, resulting in the identification of 19,770 single nucleotide polymorphism (SNPs) with a replication average of 97.4%. However, the average call rate of 56.6% indicated a large proportion (ca. 40%) of missing data. After filtering (for a replication average of >95% and a call rate of >50%) 8,478 SNPs remained. These markers had an average of 29.6% missing data which was estimated through imputation using the five nearest neighbors. To infer a position of each SNP in the *V. faba* genome, the marker sequences were mapped to the ‘Hedin/2’ reference genome (Jayakodi et al., 2023), resulting in 6,606 markers with a single mapped position. These remaining markers were evenly spread throughout the genome and over the chromosomes (**Figure 6A**) with an average SNP density of 0.5 SNP/Mbp. See **Supplementary Datasheet 2** for a full table of SNPs and sequencing data and **Supplementary Figure 3** for a linkage disequilibrium (LD) decay plot. The decay plot showed a low LD between markers, probably due to the large size of the faba bean genome and the low marker density.

*V. faba* is a partially outcrossing species, and the diversity panel was composed of accessions with varying levels of inbreeding. To obtain the best approximation of the genotype for each accession, five plants per accession were pooled before sequencing. This resulted in an average marker heterozygosity of 13.4%. In addition, there was a high frequency of rare alleles, with an average MAF of 8.5% throughout the population (**Figure 6B**). The 6,606 markers were used to estimate kinship in the population using the VanRaden method which gives a matrix where the value 0 means no genetic relatedness and 2 means complete relatedness i.e. genetically identical (**Figure 6C**). Due to the broad diversity of the panel, the relatedness amongst accessions was found to be very low. A principal component analysis (PCA) was conducted based on the genotype data (**Figure 6D**) to reveal any potential population structure. The PCA revealed that the first principal component explained only 2.55% of the genetic variance, suggesting a low degree of dependence among markers. With the different categories of advancement labelled in different colors, the PCA showed a population structure with two separated clusters of the most and least developed material (“variety” and “cultivated”, respectively). However, the mid-categories (“advanced” and “landrace”) partially overlapped with the other categories. Interestingly, the accessions in the distinctly separated group of the least developed material originated exclusively from China (see **Supplementary Figure 4** for a phylogenetic tree of the relatedness of the accessions).

### 3.6 GWAS identified markers related to ten different traits

**Figure 7:**
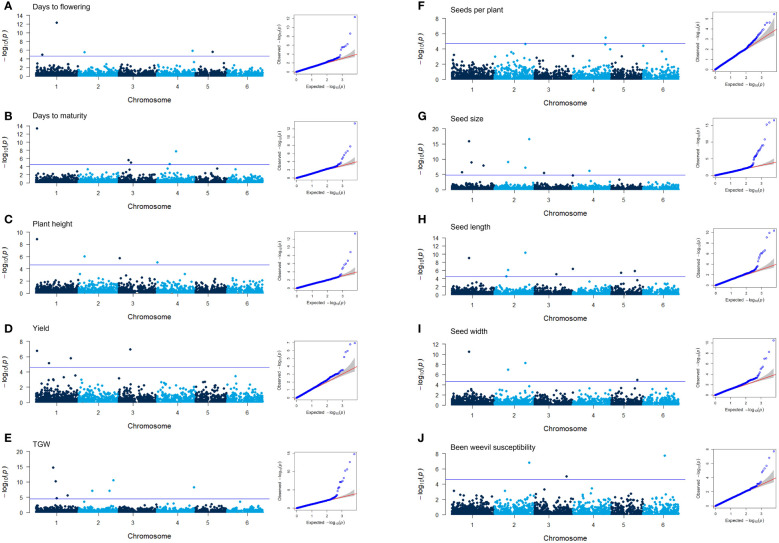
Manhattan plots of the GWAS results for 10 of the traits characterized in the diversity panel of *V. faba* and the corresponding QQ plots **(A–J)**. The blue line in the Manhattan plot signifies the false discovery rate (FDR) threshold. QQ plots showing the distribution of p-values of the markers tested for trait association in the GWAS using the BLINK model plotted against the expected distribution of p-values.

A genome-wide association study (GWAS) was conducted to identify genetic loci associated with the 12 traits assessed in the diversity panel. Using the 6,606 SNPs mapped to the faba bean genome, 51 markers were identified that showed significant associations to 10 different traits: days to flowering, days to maturity, plant height, yield, number of seeds per plant, TGW, seed size, seed length, seed width, and weevil susceptibility (**Table 2**; **Figure 7**; **Supplementary Figure 5** for starch and protein content). The quantile-quantile (QQ) plots of the GWAS with the BLINK model showed a strong association between the predicted and expected distributions of the p-values suggesting that the models appropriately accounted for the population stratification. There were no overlaps between markers for days to flowering and days to maturity. However, two markers associated with days to flowering (D13055 and D18440) were also associated with plant height and seed weight, respectively. Not surprisingly, several markers associated with different seed dimension traits showed overlaps. Of the three markers associated with weevil damage, one (D06119) was also associated with seed weight and size.

**Table 2:** Significant markers in V. faba that were associated with traits of interest identified through the GWAS, with a minor allele frequency (MAF) above 0.01.

Trait	Marker ID	Chr	Position (Mb)	Allele	p-value (log10)	MAF	Effect	Reference genome mapping Vfaba.Hedin2.R1	Sequence type	Gene annotation^a^	Homolog annotation^b^
**DTF (days)**	D10069	1S	248565604	C>T	1.00E05	0.058	2.24	1g038400	Exon	Dolichol kinase	Dolichol kinase EVAN isoform X1 [*Pisum sativum*]
	D18440	1L	857572779	T>C	4.50E13	0.138	3.12	–	Intergenic	–	–
	D13055	2	240493197	G>A	9.55E07	0.204	3.16	2g041560	Exon	Polyadenylate binding protein interacting protein	Zinc-finger BED domain-containing protein RICESLEEPER 1-like [*Arachis stenosperma*]
	D04593	4	1511466468	A>G	1.38E06	0.455	2.81	4g221040	Intron	Unknown protein	Unknown protein [*Pisum sativum*]
	D05439	5	723007937	G>T	2.42E06	0.094	1.55	5g104720	Intergenic^c^	Chromatin-remodeling ATPase, INO	Chromatin-remodeling ATPase, INO 80-like [*Pisum sativum*]
**DTM (days)**	D15934	1S	17355728	C>A	4.61E14	0.429	3.74	1g002800	Intron	Protein NRT/PTR family	Protein NRT1/ PTR FAMILY 6.1 [*Pisum sativum*]
	D08488	3	415004717	G>A	2.44E06	0.488	3.28	3g065720	Exon	Non-lysosomal glucosylceramidase	Non-lysosomal glucosylceramidase-like [*Trifolium medium*]
	D10812	3	521125812	C>G	9.58E06	0.071	1.43	3g084840	Intergenic^c^	phosphoglycerate mutase protein	phosphoglycerate mutase-like protein AT74 [*Pisum sativum*]
	D12639	4	527939461	C>T	2.16E05	0.452	1.78	4g093800	Intron	Translin-associated protein x homolog (TSNAX or TRAX)	translin-associated protein X-like [*Trifolium pratense*]
	D11891	4	808031589	G>A	1.93E08	0.429	2.20	–	Intergenic	–	
**HEIGHT (cm)**	D15935	1S	17355682	T>C	1.68E07	0.405	1.58	1g002800	Intron	Protein NRT/PTR family	Protein NRT1/ PTR FAMILY 6.1 [*Pisum sativum*]
	D13055	2	240493197	G>A	9.55E07	0.204	3.16	2g041560	Exon	Polyadenylate binding protein interacting protein	Zinc-finger BED domain-containing protein RICESLEEPER 1-like [*Arachis stenosperma*]
	D09581	3	40132566	A>T	1.84E06	0.479	8.91	3g013960	Intron	Casein kinase isoform	casein kinase 1-like protein 10 [*Vicia villosa*]
	D05633	4	5493793	C>G	9.10E06	0.469	8.84	4g001520	Intron	Alpha beta hydrolase	alpha/beta fold hydrolase [*Trifolium pratense*]
**YIELD (g/plant)**	D15935	1S	17355682	T>C	1.68E07	0.405	1.58	1g002800	Intron	Protein NRT/PTR family	Protein NRT1/ PTR FAMILY 6.1 [*Pisum sativum*]
	D01869	1S	539712640	A>G	6.84E06	0.065	1.23	1g080440	Exon	Phosphoenolpyruvate Carboxylase (PEPC)	phosphoenolpyruvate carboxylase [*Vicia villosa*]
	D18653	1L	1470877064	A>G	1.64E06	0.086	1.36		Intergenic	–	
	D00223	3	483964717	A>G	1.18E07	0.120	1.06	3g076920	Exon	Protein Pelota homolog	CDP-diacylglycerol--glycerol-3-phosphate 3-phosphatidyltransferase [*Trifolium repens*]
**SEEDS (no/plant)**	D08829	4	1393975921	G>T	3.53E06	0.094	3.28	4g204080	Exon	Fyve RHoGEF and PH domain-containing protein	FYVE zinc finger protein [*Medicago truncatula*] / vacuolar protein sorting-associated protein 27 [*Pisum sativum*]
**TGW (g)**	D08779	1S	716966083	G>A	3.35E11	0.063	1.53	1g105360	Exon	Salicylic acid glycosyl transferase, SGT homolog	protein SGT1 homolog [*Cicer arietinum*]
	D12875	1S	826078612	C>G	5.50E11	0.170	82.21	1g119840	Intron	Cytochrome c1-1 heme protein, mitochondrial	hypothetical protein TSUD_43780 [*Trifolium subterraneum*]
	D18440	1L	857572779	T>C	2.19E05	0.138	70.29	–	Intergenic	–	–
	D07779	1L	1336230743	A>T	2.39E06	0.085	86.00	1g407840	Exon	Methylthioribose phosphate isomerase	methylthioribose-1-phosphate isomerase isoform X1 [*Glycine soja*]
	D03153	2	581622870	A>G	1.08E07	0.074	1.12	2g099960	Exon	Gastric Triacylglycerol lipase	triacylglycerol lipase 2-like [*Vicia villosa*]
	D10357	2	1312826726	T>A	4.31E11	0.213	1.21	2g206480	Exon	Unknown protein	–
	D06119	2	1484092418	G>A	2.46E11	0.063	127.67	2g235160	Exon	Receptor Serine/Threonine Protein Kinase	probable serine/threonine-protein kinase PBL1 [*Vicia villosa*]
	D15752	4	1573846092	A>G	5.78E09	0.462	118.25	–	Intergenic	–	–
**SIZE (mm²)**	D18111	1L	409615010	C>T	1.84E06	0.087	14.73	–	Intergenic	–	–
	D08779	1S	716966083	G>A	3.35E11	0.063	1.53	1g105360	Exon	Salicylic acid glycosyl transferase , SGT homolog	protein SGT1 homolog [*Cicer arietinum*]
	D12875	1S	826078612	C>G	5.50E11	0.170	82.21	1g119840	Intron	Cytochrome c1-1 heme protein, mitochondrial	hypothetical protein TSUD_43780 [*Trifolium subterraneum*]
	D07779	1L	1336230743	A>T	2.39E06	0.085	86.00	1g407840	Exon	Methylthioribose phosphate isomerase	methylthioribose-1-phosphate isomerase isoform X1 [*Glycine soja*]
	D03153	2	581622870	A>G	1.08E07	0.074	1.12	2g099960	Exon	Gastric Triacylglycerol lipase	triacylglycerol lipase 2-like [*Vicia villosa*]
	D10357	2	1312826726	T>A	4.31E11	0.213	1.21	2g206480	Exon	Unknown protein	–
	D06119	2	1484092418	G>A	2.88E17	0.063	28.44	2g235160	Exon	Receptor Serine/Threonine Protein Kinase	probable serine/threonine-protein kinase PBL1 [*Vicia villosa*]
	D07466	3	401377640	C>A	3.23E06	0.491	46.80	–	Intergenic	–	–
	D15212	4	704354594	G>A	6.88E07	0.056	15.20	4g106080	Intron	Kinasine protein kin	kinesin-like protein KIN-14I [*Lotus japonicus*]
**SEED LENGTH (mm)**	D08779	1S	716966083	G>A	3.35E11	0.063	1.53	1g105360	Exon	Salicylic acid glycosyl transferase, SGT homolog	protein SGT1 homolog [*Cicer arietinum*]
	D08133	2	496103881	A>G	2.90E05	0.481	2.63	2g084080	Intron	2,3- bisphosphoglycerate dependent phosphoglycerate mutase (dPGAM)	2,3-bisphosphoglycerate-dependent phosphoglycerate mutase [*Glycine max*]
	D03153	2	581622870	A>G	1.08E07	0.074	1.12	2g099960	Exon	Gastric Triacylglycerol lipase	triacylglycerol lipase 2-like [*Vicia villosa*]
	D10357	2	1312826726	T>A	4.31E11	0.213	1.21	2g206480	Exon	Unknown protein	–
	D07408	3	916408049	G>A	8.56E06	0.085	1.06	–	Intergenic	–	–
	D05388	3	1634506611	C>A	4.14E07	0.060	1.65	3g239080	Exon	GDSL esterase lipase	GDSL esterase/lipase At1g23500-like [*Vicia villosa*]
	D04763	5	404936671	T>C	3.82E06	0.059	1.43	5g063000	Intron	Human glycosyltransferase domain-containing protein	polygalacturonate 4-alpha-galacturonosyltransferase-like [*Pisum sativum*]
	D03671	5	992670147	T>C	1.52E06	0.075	1.12	5g142480	Intron	PWWP domain isoform	hypothetical protein KIW84_031546 [*Pisum sativum*]
**SEED WIDTH (mm)**	D08779	1S	716966083	G>A	3.35E11	0.063	1.53	1g105360	Exon	Salicylic acid glycosyl transferase , SGT homolog	protein SGT1 homolog [*Cicer arietinum*]
	D03153	2	581622870	A>G	1.08E07	0.074	1.12	2g099960	Exon	Gastric Triacylglycerol lipase	triacylglycerol lipase 2-like [*Vicia villosa*]
	D10357	2	1312826726	T>A	5.25E09	0.213	0.84	2g206480	Exon	Unknown protein	–
	D08952	5	1106041368	G>A	1.07E05	0.074	0.83	5g157800	Intron	Rhomboid protein	rhomboid-like protein 15 [*Vicia villosa*]
**WEEVIL (% infested seeds)**	D06119	2	1484092418	G>A	1.56E07	0.063	8.67	2g235160	Exon	Receptor Serine/Threonine Protein Kinase	probable serine/threonine-protein kinase PBL1 [*Vicia villosa*]
	D05758	3	1356117551	C>T	9.95E06	0.118	5.99	3g196560	Exon	Protein IQ-domain	protein IQ-DOMAIN 21-like isoform X2 [*Vicia villosa*]
	D00463	6	924462796	A>T	1.89E08	0.053	10.42	6g111840	Exon	Mediator of RNA polymerase II transcription subunit	mediator of RNA polymerase II transcription subunit 16 isoform X3 [*Vicia villosa*]

a Annotation based on reference genome annotation.

b Annotation based on highest sequence similarity of legume species using blastX at NCBI (BLAST).

c Intergenic, but located less than 300bp upstream of a start codon.

### 3.7 Genomic localization of markers and candidate genes

The majority of the markers, 40 out of 51, were localized to predicted gene coding regions of the reference genome (Jayakodi et al., 2023). Annotations for these genes were derived from the reference genome and, for confirmation purposes, from genes showing the highest sequence similarity in legume species (**Table 2**). In most cases, these annotations were consistent with each other. The candidate genes encode proteins with functions in central biosynthetic pathways, DNA/RNA metabolism, and transcriptional regulation or as hormone and metabolite transporters.

## 4 Discussion

### 4.1 Importance of diversity panels in pre-breeding

In this work, we assembled a diversity panel of 220 faba bean accessions including a wide range of geographical origins and breeding advancements. This panel was characterized through genotyping and field phenotyping under Nordic climate conditions, in this case a warm-summer humid continental climate (Arnfield, 2023). We genotyped our diversity panel using the genotyping-by-sequencing (GBS) approach DArTseq to identify single nucleotide polymorphism (SNP) markers (Baloch et al., 2017). Based on this genotype data it became evident that the accessions exhibited a very low degree of relatedness as supported by the kinship matrix and the principal component analysis (PCA) indicated a low dependence among markers. The comprehensive phenotypic characterization of this diversity panel through field trials conducted over two consecutive years revealed substantial variation in key agronomic and seed quality traits (days to flowering, days to maturity, plant height, bean weevil susceptibility, seed yield, thousand grain weight, number of seeds per plant, seed size, seed width, seed length, protein content, and starch content). The large variation in seed size of *Vicia faba* is well known and has historically led to its categorization into subspecies major (large‐seeded), equine (medium‐seeded), minor (small‐seeded), and paucijuga (small‐seeded) types. However, due to the phenotypic continuity rather than distinctness, and their reproductive compatibility, these types are today regarded botanical groups of the same species (Maalouf et al., 2018; Jayakodi et al., 2023). Several of the characterized traits showed high broad-sense heritability values, indicating a strong additive genetic component. The heritability of seed size traits in our study was very high and similar to those seen in previous work with values close to 1.00 (Skovbjerg et al., 2023). Our results from characterization of a diversity panel of faba bean present a valuable resource for identifying germplasm with desirable traits for breeding purposes. It can be noted that extending the characterization of this diversity panel across multiple years and locations would be beneficial to confirm the genetic impact on specific traits.

The phenotype and genotype data were further used in a genome-wide association study (GWAS) to identify marker-trait associations. Our SNP marker data comprised a high rate of rare alleles (**Figure 6B**). It should be noted that the choice of MAF threshold affects the power of GWAS and is commonly set to 0.05 (Alqudah et al., 2020). However, to capture rare alleles in diverse panels which can be of interest as targets for further genetic and biological studies, the MAF threshold in the GWAS can be lowered to not exclude any potential markers of interest (Stanton-Geddes et al., 2013; Arkwazee et al., 2022; Kim et al., 2022). In the current study, the MAF was therefore set to >0.01, however, it is noteworthy, that the markers identified as significantly associated with traits all had a MAF exceeding 0.05. While rare alleles could lead to spurious associations in GWAS as they are present in very few individuals, it is worth noting that some rare alleles may control important traits. Therefore, rare alleles in GWAS require careful attention (Gibson, 2012). For example, rare alleles were found to be associated with important agronomic traits in wheat (Jaiswal et al., 2016), and grain size and yield in rice (Hu et al., 2015). It is important that the associated markers with rare alleles identified in this study, are validated in different genetic backgrounds or larger populations before being used in marker-assisted selection to improve agronomic and yield traits in faba bean. Overall, a less diverse panel (in terms of level of advancement, germplasm origin, and relatedness) or a larger number of accessions would have been beneficial to capture a more extensive set of associated markers, since these factors impair the genetic analysis of the traits of interest (Torkamaneh and Belzile, 2022). However, the aim of this study was to identify novel loci of interest for trait screening instead of the development of markers for direct implementation in genomic breeding of advanced lines.

### 4.2 Phenotypic breeding advancement and target traits for Nordic regions

For the Nordic region which is characterized by a short growing season, the relatively long growth period of faba bean as compared to other spring-sown crops emphasizes the significance of prioritizing earliness traits as a key breeding target (Stoddard and Hämäläinen, 2011). Nevertheless, we showed that previous breeding advances in the faba bean germplasm have indeed resulted in notable improvements such as higher yields and a greater number of seeds per plant, but also a later onset of flowering and maturity. It is expected that the modern varieties were higher yielding, considering that most of the modern varieties in our diversity panel were developed for central or southern Europe, a climate region fairly similar to the latitude of our field trials, as compared to the wider geographical origin of the less improved material. Furthermore, given that the growing season is relatively long in southern and central Europe and that earliness traits have thus not been prioritized in breeding programs of faba bean in this region, it is not surprising that modern varieties in our diversity panel showed a later onset of flowering and maturation.

Our phenotypic correlation studies based on all accessions in the diversity panel showed a significant positive relationship between late maturity and higher yields, and a higher number of seeds per plant. However, our observation of broad variation in earliness traits, with more than two weeks difference in days to flowering as well as in days to maturity, signifies that relevant germplasm is available for breeding aimed at earlier varieties. The accessions showing the earliest flowering originated from diverse geographical regions including the Nordic latitudes, and represented several landraces (for example Anuksen Kanta, Brottby, Grebo, and Gubbestad) but also vegetable varieties available for hobby growers (such as Crimson flowered, Hangdown, and Robin Hood). Our pairwise correlation analysis revealed a strong correlation between early flowering and large seed size, of which the latter is usually not a desirable trait for cultivation on larger scale, due to their incompatibility with sowing and harvest machines and lower seed amplification factor. However, among the accessions in our diversity panel it is possible to identify accessions combining early flowering (less than 57 days to flowering), reasonable seed size (thousand-grain weight (TGW) between 400-700 g) and high yield (>10 g/plant) such as Cervci, Talia, FAB5138, FAB6776, Brottby, Jygeva, Gubbestad, and Habas de Beck).

### 4.3 Food quality aspects of faba bean seeds are important breeding targets

The increased application of faba bean for human consumption is relying on its nutritional composition (Martineau-Côté et al., 2022). In our work, post-harvest seed quality aspects were characterized alongside the agronomic traits, shedding light on the variability of traits such as protein and starch content of, 25-38% and 21-38% by seed dry weight, respectively. These ranges are in line with reports from other faba bean studies (Martineau-Côté et al., 2022; Zhao et al., 2023). Our findings further indicated that there was no observed yield penalty for increased seed protein content, under our experimental field conditions. This stands in contrast to what is well-known for wheat, for example, where seed protein content is negatively correlated with yield (Oury and Godin, 2007; Laidig et al., 2017). On the other hand, in pea, both negative and positive correlations between seed protein content and yield have been reported (Daba and Morris, 2022). Our results showed a negative correlation between protein and starch content in faba bean, which has also been observed in pea (Daba and Morris, 2022), implying that breeding for increased protein content potentially leads to a reduction of starch. Furthermore, our results showed that TGW was negatively correlated to both protein and starch content, indicating that smaller seeds have a higher protein and starch content.

In recent times broad bean weevil (*Bruchus rufimanus*) infestation of faba bean seeds has emerged as a severe pest (Huber et al., 2023). Despite the rather strict quality standards in place with a maximum acceptance of 3% infested seeds for human consumption and less than 10% for animal feed, broad bean weevil resistance remains elusive, and the understanding of chemical attractors associated with the pest is still limited (Dell’Aglio and Tayeh, 2023). The overall weevil infestation was high in our diversity panel. However, our data uncovered a gradient among accessions, suggesting varying levels of susceptibility to bean weevil damage. This observation aligns with prior studies that have highlighted varietal differences in susceptibility to bean weevil damage (Carrillo-Perdomo et al., 2019; Segers et al., 2022; Dell’Aglio and Tayeh, 2023). Based on the results in our study and in previously published data (Dell’Aglio and Tayeh, 2023), selecting for traits such as low plant height, late flowering and maturation and small seed size could be a possible approach to mitigate weevil infestation, since they were all negatively correlated to weevil damage of seeds. However, it should be considered that the early flowering accessions can act as catch crops. In fact, Dell’Aglio and Tayeh (2023) suggested that earlier flowering plants may offer pods earlier while larger seeds provide more food for the larvae, potentially contributing to a higher susceptibility to weevil infestation. Other studies have shown that bean weevil damage reduces yields in faba bean (Carrillo-Perdomo et al., 2019; Segers et al., 2022) but our study did not show this correlation. It should be noted that our plant material exhibited a high diversity and multiple factors with small effects probably contribute to the varying degree of bean weevil susceptibility. Further studies are necessary to develop effective strategies for managing and overcoming this pest in faba bean production.

### 4.4 Novel markers for ten different traits identified in candidate genes

Several reports on different crops including legumes have shown the potential of using DArTseq to determine population structures and associating genetic markers with different traits (Akbari et al., 2006; Raman et al., 2014; Aznar-Fernández et al., 2020; Alemu et al., 2022). By using DArTseq in this population we could identify 6,606 SNP markers evenly distributed in the faba bean genome, and 51 of those markers were identified to be associated with ten of the characterized traits through our GWAS. Among these traits, seed size characters and TGW were predominantly represented in the list of markers, along with yield, number of seeds per plant, plant height, days to flowering, days to maturation, and bean weevil damage.

Three of the markers associated with days to flowering were localized to predicted genes with homologs in other plant species encoding proteins that regulate flowering; namely dolichol kinase, polyadenylate binding protein interacting protein, and chromatin-remodeling ATPase INO (Zhang et al., 2015; Cho et al., 2017; Wang et al., 2019). Those markers have not been reported in previous marker-trait studies on faba bean targeting flowering traits (Aguilar-Benitez et al., 2021; Skovbjerg et al., 2023). The chromatin-remodeling ATPase INO80 has a prominent role in the regulation of the key flowering repressor *FLOWERING LOCUS C*, and Arabidopsis *ino80* mutants show a delayed onset of flowering (Zhang et al., 2015). Interestingly, the marker identified in our study was localized not within but 209 bp upstream of the predicted gene, with a high probability of being part of its regulatory region. Days to flowering is physiologically connected to days to maturation, and our phenotype data showed a strong positive correlation between them. In our study, markers associated with days to maturation were localized to predicted genes for which homologs in other plant species have functions in plant hormone transport, sphingolipid metabolism, glycolysis, and RNA/DNA metabolism (Zhao and Assmann, 2011; Chiba et al., 2015; Gupta et al., 2017; Dai et al., 2020).

One of the four markers associated with plant height was also associated to yield, and only 46 bp away from a marker associated with days to maturation, all localized to the same predicted gene involved in hormone transport. Another marker associated with plant height was also associated with days to flowering. The genetic markers linked to plant height as identified by Skovbjerg et al. (2023) were not identical to the markers in our dataset. Two of the markers associated to seed yield in our study were localized to predicted genes with homologs in other plant species being involved in glycolysis and lipid metabolism. One of them encodes a phosphoenolpyruvate carboxylase that has an important role in carbon and nitrogen metabolism, and has recently been linked to seed yield in Arabidopsis (Shi et al., 2015; Feria et al., 2022).

Of the six markers associated with TGW, one was localized to a predicted gene encoding a salicylic acid glycosyl transferase. Salicylic acid is a phytohormone that is usually connected to functions in biotic and abiotic stress response but has, interestingly, also been shown to regulate the number of flowers and pods in chickpea (Li et al., 2022). Another marker was localized to a predicted gene for which the homolog in Arabidopsis encodes methylthioribose phosphate isomerase shown to be involved in promoting flower and seed development (Zierer et al., 2016). As expected, a large number of the markers associated with TGW were also associated with the seed dimension traits area, length, and width. Skovbjerg et al. (2023) previously identified similar marker overlaps for various seed dimension parameters in faba beans. However, none of the genes mapped by their markers were present in our dataset. The three markers found by Zhao et al. (2023) did not match our markers for TGW and seed size features. Historically, the selection of faba bean likely relied solely on visual traits of the seeds’ morphology. Thus, the discovery of genetic markers related to seed characteristics might be promising for further breeding endeavors.

One of the three markers associated with bean weevil damage was also associated with seed size. In fact, a correlation between seed size and bean weevil susceptibility was observed in our phenotype data where larger seeds had a higher ratio of bean weevil damage. The second marker was localized to a predicted gene for which an Arabidopsis homolog encodes an IQ domain protein with a function in calcium ion signaling (Fischer et al., 2013), which is important in biotic stress response (Aldon et al., 2018). The third marker was localized to a gene homologous to a subunit of the mediator complex of RNA polymerase II which in Arabidopsis is involved in transcriptional responses to cell wall defects (Buendía-Monreal and Gillmor, 2016). These functions can potentially be involved in the response to bean weevil attacks.

Despite the large variation observed in seed protein and starch content in our diversity panel, the GWAS did not reveal any markers associated with these traits. Recently, Zhao et al. (2023) revealed 22 markers associated with seed quality traits, including seed protein, starch, and lipid content. It is noteworthy that those markers were identified using a genotyping platform with a 130 K SNP chip based on RNA data from flower and leaf tissue but not from seed tissue (Wang et al., 2021).

The absence of overlap between our associated markers with earlier reported markers in faba bean is not entirely surprising, when taking into consideration the species vast genome and the relatively few available studies on faba bean so far. Further, our diversity panel presents a heterogenous population with a broad genetic base compared to the more narrow genetic bases of other studies.

### 4.5 Challenges with genotyping of faba bean being a partly outcrossing species

Genotyping by sequencing (GBS) approaches has the potential to identify novel SNP at a high density which can improve genomic analyses such as GWAS, genomic prediction and QTL mapping (Meuwissen and Goddard, 2010; Druet et al., 2014; Alipour et al., 2019). However, many GBS approaches suffer from an abundance of missing data due to low sequencing depth which can significantly lower the number of usable SNPs. DArTseq relies on a combination of restriction enzymes for the digestion of the genome prior to the sequencing of the resulting segments (https://www.diversityarrays.com/). One explanation for the large proportion of the missing data, of the original 19,770 SNP markers, could be that no SNP call could be produced for segments in the accessions where the upstream restriction enzyme site was not present and therefore not sequenced. Through the DArTseq method it was possible to identify a large amount of polymorphisms in the genome that could potentially be used for development of marker chips, however for the genotyping of multiple individuals in a large diverse population, sequencing methods based on digestion with restriction enzymes might not be preferable.

Increasing sequencing depth has the potential to lower the amount of missing data through higher repeatability in marker sequence reads and coverage of the genome, simply through the increase of reads per sample but at higher costs. Instead, improvement of the data through imputation of the missing values could increase the usability of the genetic data. Using close neighbors to impute missing genotyping data from GBS platforms is an efficient strategy which increases the accuracy of downstream genetic analyses (Gamal El-Dien et al., 2015). In this study the proportion of missing data was 29.6% after filtration steps, which was imputed using the five closest neighbors. While our study successfully identified 51 novel markers associated to ten different traits, it is important to acknowledge the potential impact of the genotyping challenges and the approach of genotyping pooled individuals. The majority of associated markers were located on chromosome 1, the largest among the six chromosomes. Interestingly, no markers were identified on chromosome 6, despite the generally even distribution of the 6,606 SNP markers across the faba bean genome. The relatively low marker coverage on individual chromosomes, considering the vast 13 Gb genome of faba bean, may account for this phenomenon. Increasing the sequencing depth could potentially have resulted in a higher density of informative markers, enhancing the dataset’s comprehensiveness and robustness. This, in turn, might have facilitated the identification of more markers associated with traits of interest.

As mentioned earlier, the relatively high proportion of missing data could partly be addressed through imputation. However, the partly outcrossing nature of faba bean, leading to increased heterozygosity within accessions, raises concerns about accurately determining genotypes. To account for this, genotyping of each accession in the panel was performed on five pooled individuals aiming to determine the genotype of that ‘population’ rather than of individuals. In general, to capture a representative sample of the genetic variation within a population, genotyping a larger number of individuals for each accession would have been beneficial. Further, potential misclassification of genotypes as homozygotes or heterozygotes for the SNPs, possibly caused by handling SNP data from pooled individuals (in which the individuals’ genotypes cannot be properly identified), could influence the interpretation of marker-trait associations. For example, if minor alleles are distributed evenly within, rather than among accessions, this might wrongly infer homozygotes at many loci. However, rare alleles present at a low frequency within an accession might only contribute to a small proportion of the genetic effect on the resulting phenotype. Therefore, while the novel markers from our study show promise in being associated with target traits and to genes with predicted functions in key developmental processes in plants, further evaluation is needed to assess their reliability and practical utility in breeding programs.

## 5 Conclusions

Recent breeding efforts on faba bean are scanty in comparison to other legumes, such as soybean or pea (Rubiales et al., 2021). However, the recently published reference genome of faba bean (Jayakodi et al., 2023) has opened new possibilities for exploring the genetics of faba bean with significantly higher resolution and efficiency and how this can be used in future plant breeding. To establish a sustainable supply of locally produced green proteins, concerted breeding efforts are crucial for faba bean targeting different geographical regions and leveraging genetically informed breeding techniques. To address these challenges, plant breeders can harness the vast genetic diversity available in faba bean germplasm collections held in genebanks (Duc et al., 2010; Rubiales et al., 2021). This necessitates an investment in pre-breeding, which involves the thorough characterization of germplasm through both phenotypic and genotypic assessments, as exemplified by the findings presented in this study. The results from this study contribute to the growing pool of phenotypic and genotypic data on faba bean, which provides a valuable resource for developing efficient breeding strategies for faba bean.

## Data availability statement

The original contributions presented in the study are included in the article/**Supplementary Material**, further inquiries can be directed to the corresponding author/s.

## Author contributions

HO: Data curation, Investigation, Methodology, Validation, Visualization, Writing – original draft, Writing – review & editing. JÅ: Data curation, Investigation, Methodology, Validation, Visualization, Writing – original draft, Writing – review & editing. AlC: Conceptualization, Validation, Writing – review & editing. DB: Conceptualization, Writing – review & editing. CH: Conceptualization, Investigation, Methodology, Validation, Writing – review & editing. AaC: Conceptualization, Investigation, Methodology, Supervision, Validation, Writing – review & editing. ÅG: Conceptualization, Data curation, Funding acquisition, Investigation, Methodology, Project administration, Supervision, Validation, Visualization, Writing – original draft, Writing – review & editing.
