# Supplementary material for: Novel SNP markers for flowering and seed quality traits in faba bean (Vicia faba L.): characterization and GWAS of a diversity panel
Source: Front Plant Sci. 2024 Mar 6;15:1348014. doi: 10.3389/fpls.2024.1348014 (PMC10950902; doi:10.3389/fpls.2024.1348014)
Supplement: Supplementary file 3 [file DataSheet_3.docx]

Supplementary Material

# Supplementary Data

**Supplementary Dataset 1**. Passport data for accessions in the diversity panel (accession IDs, groupings for type, region and origin), phenotypic data (agronomic and seed quality traits), weather data, soil conditions, and field management. (Excel spreadsheet)

**Supplementary Dataset 2.** Table of the 6,606 SNPs and sequencing data. (Excel spreadsheet)

# Supplementary Figures and Tables


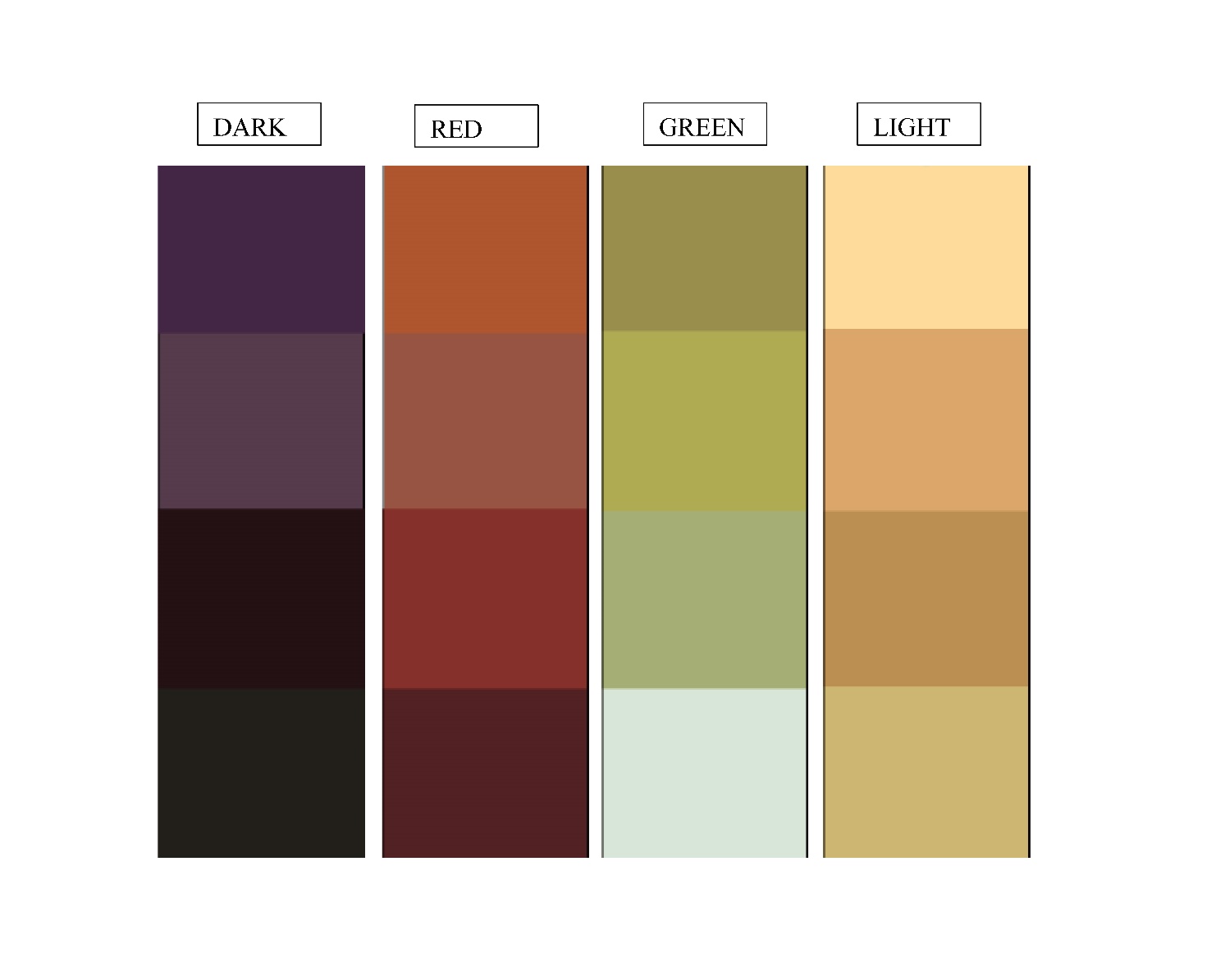


Supplementary Figure 1. Faba bean seed testa color classification. The reference colors used in each category were obtained by sampling photos of the seeds with the color picker tool (Affinity Photo software).

**
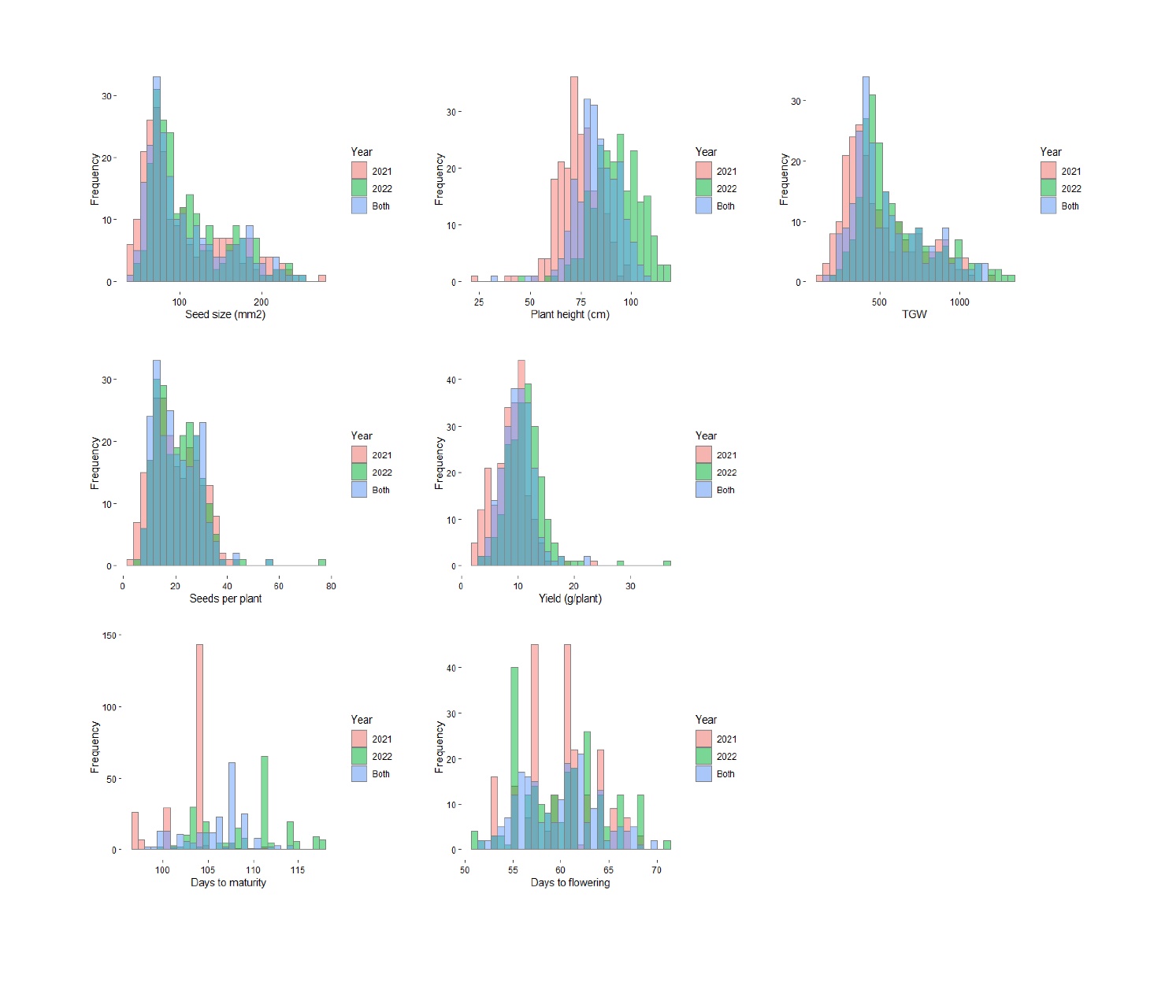
**

**Supplementary Figure 2.** Histograms showing the distribution of BLUE values for each trait evaluated in the faba bean diversity panel for the two different years of field trials. Values for 2021 shown in red bars, 2022 in green bars, and both years in blue bars.


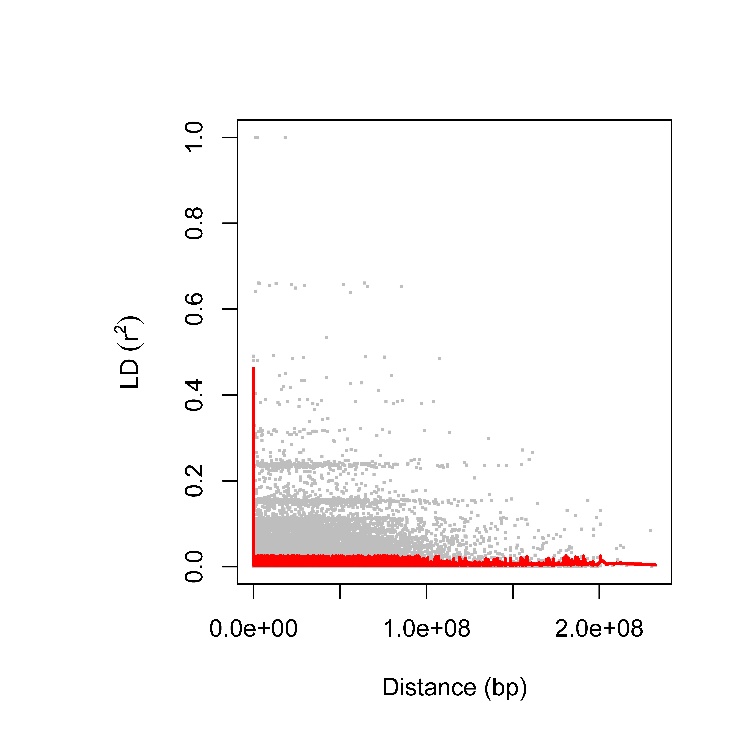


**Supplementary Figure 3.** Scatter plot showing the linkage disequilibrium (LD) decay across the chromosomes for the 187 accessions of the diversity panel. The genetic distance in base pairs (bp) is plotted against the LD estimate (r^2^) for pairs of SNP markers.


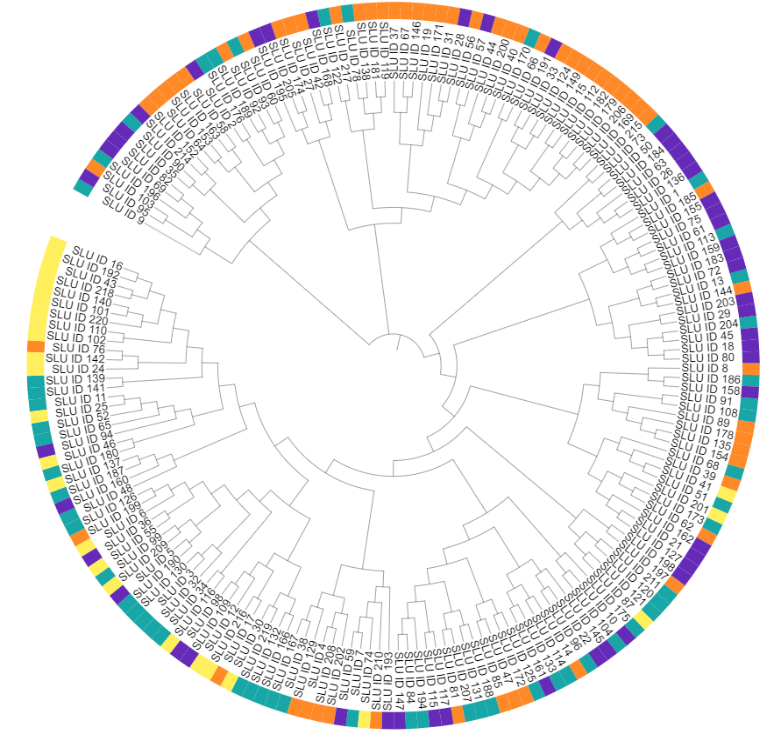

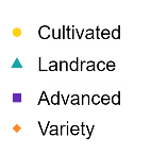


**Supplementary Figure 4***.* Phylogenetic tree of the relatedness of the accessions based on genotype data and with accessions color coded according to the level of advancement. For passport data for each accession see IDs in Supplementary Dataset 1.


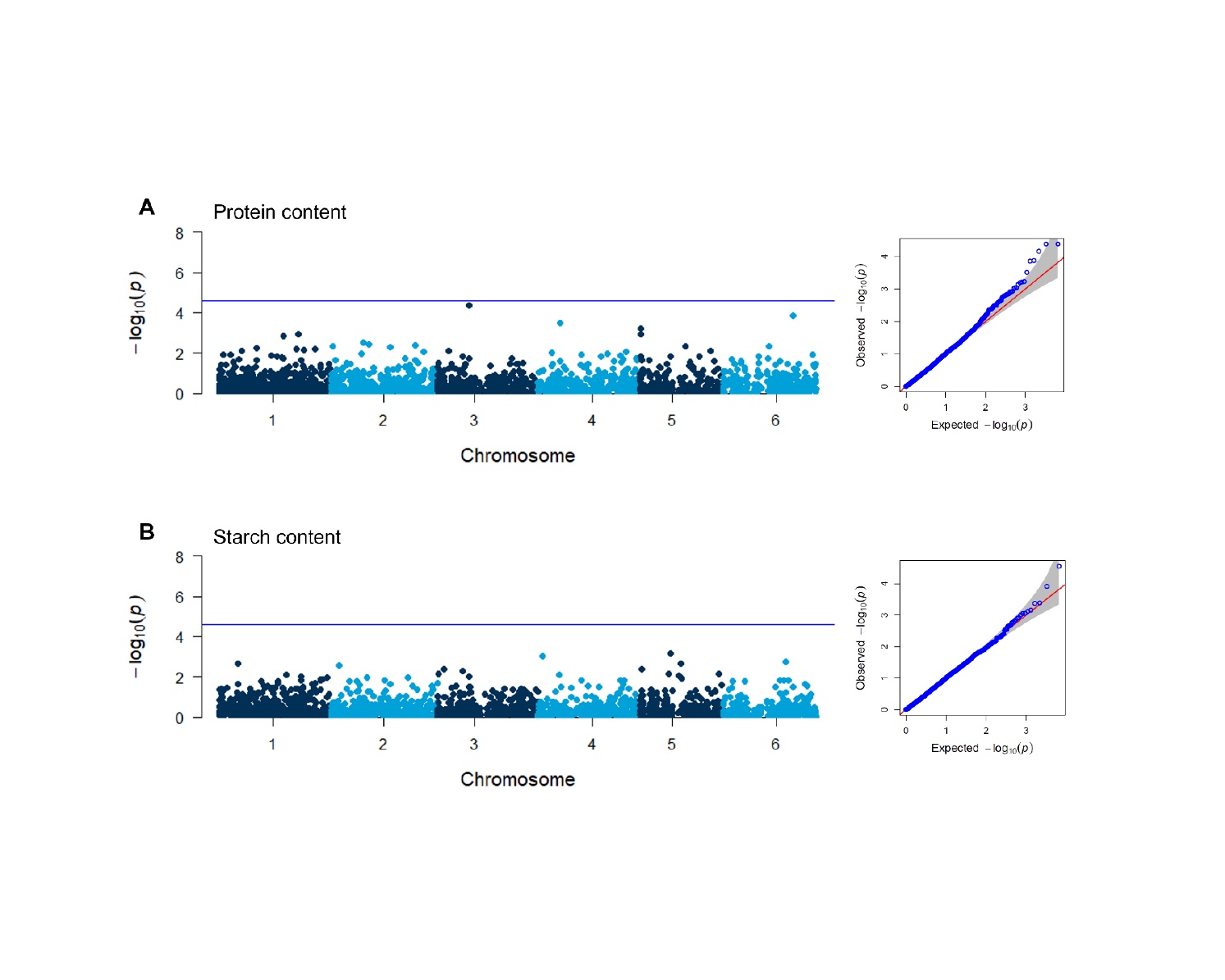


Supplementary Figure 5. Manhattan plots of the GWA studies results for the traits protein (A) and starch content (B) in the diversity panel of faba bean and their corresponding QQ plots. The blue line in the Manhattan plot signifies the FDR threshold. QQplots showing the distribution of p-values of the markers tested for trait association in the GWA study using the BLINK model plotted against expected distribution of p-values.

**Supplementary Table 1.** Pairwise correlations between traits characterized in the diversity panel based on phenotypic values, described by Pearson correlation coefficient. Number of stars indicates the significance based on ANOVA t-test with p < 0.001: ***, p < 0.01: **, p < 0.05: *. WEEV = proportion of seeds infested by been weevil (%); TGW= thousand grain weight (g) ; SIZE = seed size as area (mm²); FLOW = time from sowing to flowering (days); MAT = time from sowing to maturity (days); HEIGHT = plant height (cm); YIELD = seed weight per plant (g); SEEDS = number of seeds per plant, PROT = seed protein content by dry weight (%), STCH = seed starch content by dry weight (%).

|  | FLOW | HEIGHT | MAT | TGW | YIELD | SIZE | WEEV | SEEDS | PROT |
| --- | --- | --- | --- | --- | --- | --- | --- | --- | --- |
| HEIGHT | 0.37 *** |  |  |  |  |  |  |  |  |
| MAT | 0.63 *** | 0.49 *** |  |  |  |  |  |  |  |
| TGW | -0.50 *** | -0.19 ** | -0.02 |  |  |  |  |  |  |
| YIELD | 0.03 | 0.49 *** | 0.53 *** | 0.23 *** |  |  |  |  |  |
| SIZE | -0.53 *** | -0.28 *** | -0.11 | 0.99 *** | 0.16 * |  |  |  |  |
| WEEV | -0.68 *** | -0.52 *** | -0.60 *** | 0.55 *** | -0.19 ** | 0.61 *** |  |  |  |
| SEEDS | 0.50 *** | 0.47 *** | 0.38 *** | -0.74 *** | 0.40 *** | -0.77 *** | -0.66 *** |  |  |
| PROT | 0.12 | -0.07 | 0.06 | -0.27 *** | -0.07 | -0.27 *** | -0.26 *** | 0.27 *** |  |
| STCH | 0.17 * | 0.48 *** | 0.14 * | -0.24 *** | 0.17 * | -0.30 *** | -0.21 ** | 0.26 *** | -0.38 *** |
